# The Utility of a Flexible Fluorescence-Cystoscope with a Twin Mode Monitor for the 5-Aminolevulinic Acid-Mediated Photodynamic Diagnosis of Bladder Cancer

**DOI:** 10.1371/journal.pone.0136416

**Published:** 2015-09-02

**Authors:** Hideo Fukuhara, Mitsuhiro Kureishi, Takeo Khoda, Katsushi Inoue, Tohru Tanaka, Kohei Iketani, Masahiro Orita, Keiji Inoue, Taro Shuin

**Affiliations:** 1 Department of Urology, Kochi Medical School, Kohasu, Oko, Nankoku, Kochi 783–8505, Japan; 2 R&D Center, HOYA Corporation, 3–3–1 Musashino, Akishima-shi, Tokyo 196–8510, Japan; 3 SBI Pharmaceuticals Company, Ltd., 1–6–1 Roppongi, Minato-ku, Tokyo 106–6020, Japan; Massachusetts General Hospital, UNITED STATES

## Abstract

**Objectives:**

To evaluate the diagnostic value of a new photodynamic diagnosis (PDD) system using 5-aminolevulinic acid (ALA) for the diagnosis of bladder tumors. To validate whether false-positive findings caused by tangent effects in PDD can be resolved, we compared diagnostic accuracies between the new PDD system and a conventional PDD system.

**Patients and Methods:**

Among 30 transurethral bladder biopsies, 15 cases received ALA-PDD using rigid fluorescence cystoscopy (conventional PDD system), and flexible fluorescence cystoscopy with a twin mode monitor (new PDD system) was used in a separate set of 15 cases. To evaluated the usefulness of ALA-PDD, diagnostic accuracies were retrospectively compared between the conventional PDD system and the new PDD system.

**Results:**

Of 207 specimens from 30 cases, we obtained 110 specimens using the conventional PDD system and 97 specimens using the new PDD system. Of these samples, we selected 30 distal bladder specimens each from both the conventional PDD system and the new PDD system. The overall sensitivity, specificity and false-positive rate for the new PDD system were 100%, 82.6%, and 17.4%, respectively. Those of the conventional PDD system were 83.3%, 66.2% and 33.8%, respectively. The overall false-positive rate of the new PDD system improved to 16.4% when compared with the conventional PDD system. Furthermore, the false-positive rate of the new PDD system in distal bladder samples improved to 11.8%. The overall AUC of the new PDD system was significantly greater compared with that of the conventional PDD system (*P*<0.05). We obtained similar significant results in the distal bladder samples (*P*<0.05). All procedures were well tolerated by all patients without any severe adverse events.

**Conclusion:**

Flexible cystoscopy had a significantly higher specificity and improved incidence of tangent effects when compared with conventional methods. This preliminary study suggests that the new PDD system using 5-aminolevulinic acid may be more useful than the conventional PDD system.

## Introduction

Bladder cancer is the second most common urological cancer and has an annual incidence of over 60,000, 130,000, and 17,000 in Europe, the United States, and Japan, respectively [1–3]. Approximately 70% of cases involve non-muscle invasive bladder cancer treated with transurethral resection of the bladder tumor (TURBT) [4].

However, non-muscle invasive bladder cancer has a high recurrence rate after TURBT. It is thought that residual lesions, such as minute or flat lesions, significantly contribute to the high recurrence rate. Photodynamic diagnosis (PDD) using 5-aminolevulinic acid (ALA) provides good visualization for these minute and flat lesions and improves diagnostic accuracy [5–12].

We previously reported the feasibility of ALA-PDD for bladder cancer diagnosis [13]. ALA-PDD significantly improved the diagnostic accuracy and intraoperative detection of bladder cancer, particularly for flat lesions such as dysplasia and CIS. Compared with observation under white light, ALA-PDD provides a relatively high sensitivity for bladder cancer diagnosis. However, this method produced many false-positive findings in the distal bladder (trigone and neck portion of the bladder). The specificity in these portions was significantly lower than that in other portions of the bladder. The main reason for false-positive findings in the trigone and neck portion is that red-fluorescence is enhanced in the direction of the tangent, even if the tissue is normal. The phenomenon is called the tangent effect. However, it is difficult to observe the bladder neck with a rigid scope in a perpendicular direction. This tangent effect is an important problem for false-positive findings in ALA-PDD.

However, a flexible scope can be used directly on the bladder neck tissue and therefore prevent the tangent effect. In new PDD system, a flexible scope was used to observe the bladder cervix along with a processor (SAFE-3000, PENTAX, HOYA Co, Tokyo) that can simultaneously display both a white light image and a fluorescent image on a video monitor (twin mode). With systems where only one image (white or fluorescent light) can be displayed at a time, surgeons must compare the two images by memory when switching back and forth. Utilizing twin mode, both images can be compared on the same monitor, which is expected to improve diagnostic accuracy.

In previous reports, no study has examined the false-positives caused by the tangent effect during ALA-PDD in bladder cancer. In this study, we retrospectively evaluated the feasibility of ALA-PDD using the SAFE-3000 system (new PDD system) and whether the flexible cystoscopy improves false-positive findings and diagnostic accuracy in the trigone and neck portion of bladder.

## Patients and Methods

### Ethics Statement

This study was carried out with approval from the Ethics Committee of Kochi Medical School (study number; ERB-00026). Written informed consent was received and recorded from all participants prior to enrollment in this study. All participants were informed about the potential treatment efficacy and adverse events, such as vomiting, skin photosensitivity and elevation of serum ALT/AST, in accordance with the Common Terminology Criteria for Adverse Events version 3.0. A total of 30 cases, 15 ALA-PDD cases using flexible fluorescence cystoscopy and twin mode monitor of SAFE-3000 system (new PDD system) and 15 ALA-PDD cases using rigid fluorescence cystoscopy (conventional PDD system) as a historical control, were enrolled in the study [S1 file].

### Patients and study design (Table 1/Fig 1)

ALA-PDD was performed in all 30 cases, of which 23 were men and 7 were women, with a median age of 68.9 (49–81) years. Sixteen cases were primary, and 14 cases were recurrent. Characteristics of the patients treated using the new PDD system or the conventional PDD system are shown in Table 1. To determine the feasibility of the new PDD system, the diagnostic accuracy based on the area under curve (AUC) from the new PDD method was retrospectively compared with that of the conventional PDD system as a historical control. The results were also compared with those obtained by conventional white light examination. There were no statistically significant differences among patients with regards to tumor characteristics. All patients were followed up for at least 3 years. Follow-up cystoscopies were performed every 3–6 months.

**Fig 1:**
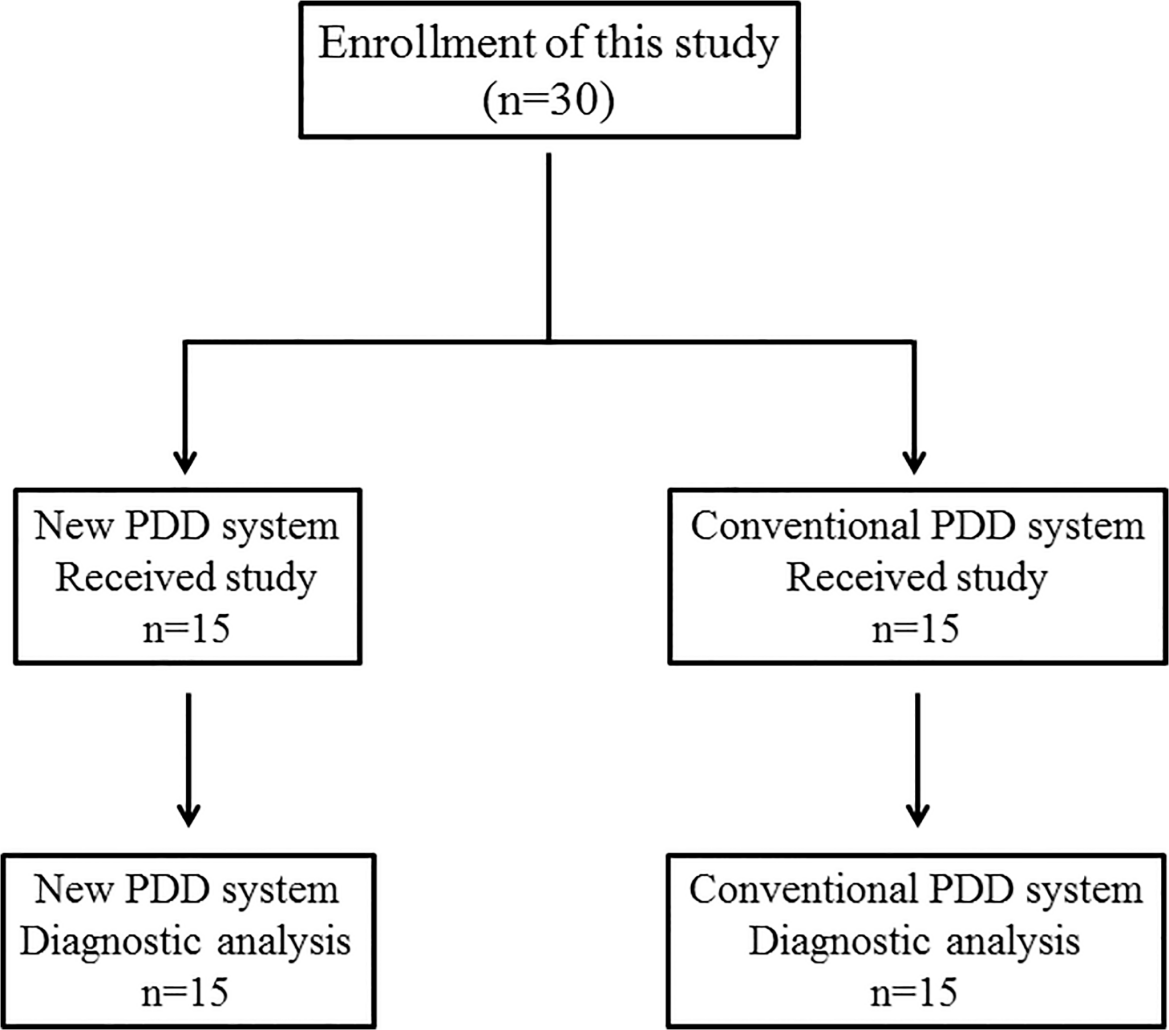
Flow chart of this study on new PDD system and conventional PDD system in the diagnosis of bladder cancer

**Table 1:** Patient characteristics.

Variable		Conventional PDD system	New PDD system
Patients		15	15
Examination period		Jan’09–Dec ‘09	Dec’11-July’12
Age (yr)	Mean	68.9	68.4
	Range	49–81	50–87 *P* = *0*.*90* ^*a*^
Sex	Male	13	10
	Female	2	5 *P* = *0*.*20* ^*a*^
Past history	Primary case	10	8
	Recurrent case	5	7 *P* = *0*.*46* ^*a*^
Prior therapy	TUR-Bt	4	2
	TUR-Bt + BCG	1	5 *P = 073* ^*a*^
Tumor stage	No malignancy	2	6
	pT is	2	1
	a	8	4
	1	3	2
	2	0	1
	3	0	1 *P* = *0*.*32* ^*b*^

^a^ Fisher exact test(2x2)

^b^ Chi-square test

### Administration of 5-aminolevulinic acid

We applied ALA as a photosensitizer for ALA-PDD. ALA hydrochloride (COSMO BIO, Tokyo, Japan) was dissolved in 50 ml of 5% glucose and 40 ml of 8.4% sodium hydrogen carbonate (NaHCO_3_). Patients were orally administered 1.0 g of ALA solution two to three hours prior to intraoperative surveillance and before anesthesia.

### Photodynamic diagnosis system (new PDD system)

A fluorescence endoscope consisting of a processor (SAFE-3000) and a flexible cystoscope (EB-1970AK, PENTAX) was used as the new PDD system. A system diagram of the processor contains a xenon lamp as a white light source (300 W) with a semiconductor laser (wavelength of 408 nm) with a power at the top of the scope of 20 to 40 mW. The wavelength of the laser is close to that of the absorption maximum of PpIX at 405 nm. A series of processes (absorption by PpIX, excitation of PpIX, and fluoresce from PpIX) can then be detected with a high efficiency. The spectrum of the fluorescence has a broad peak at approximately 635 nm; fluorescence images can be observed with a charge coupled device image processor (CCD). A filter is mounted on the CCD in order to reduce the strong blue light reflected by organ surfaces. A white light image and a fluorescent image are simultaneously displayed side-by-side on a monitor (twin mode). The side-by-side image is composed of a white light image captured within the first 1/60 second and a fluorescent image captured in the next 1/60 second. In the first 1/60 second, white light is emitted through a shutter that opens for 1/60 second. In the next 1/60 second, a blue laser light is emitted by switching on the laser chip for 1/60 second. A white light image from the first 1/60 seconds and a fluorescent image from the following 1/60 second are held in a memory chip, and each image is simultaneously displayed for 1/30 second on the monitor to produce video images at 30 frames/second. The diameter of the scope is 6.3 mm at the top and 2.8 mm at the channel. The bending angle at the top is 130 to 180 degrees. It should be noted that the EB-1970AK scope is designed for use in the bronchus and was used for bladder cancer observation in this study.

### Intraoperative procedure (Fig 2)

Transurethral observation of the bladder was performed under white light and fluorescence light guidance. The specimens with fluorescent emission or with suspicious abnormalities under white light-guided observation were systematically harvested from 7 bladder regions (left, posterior, right walls, trigone, bladder neck and prostatic region of the urethra.)

**Fig 2:**
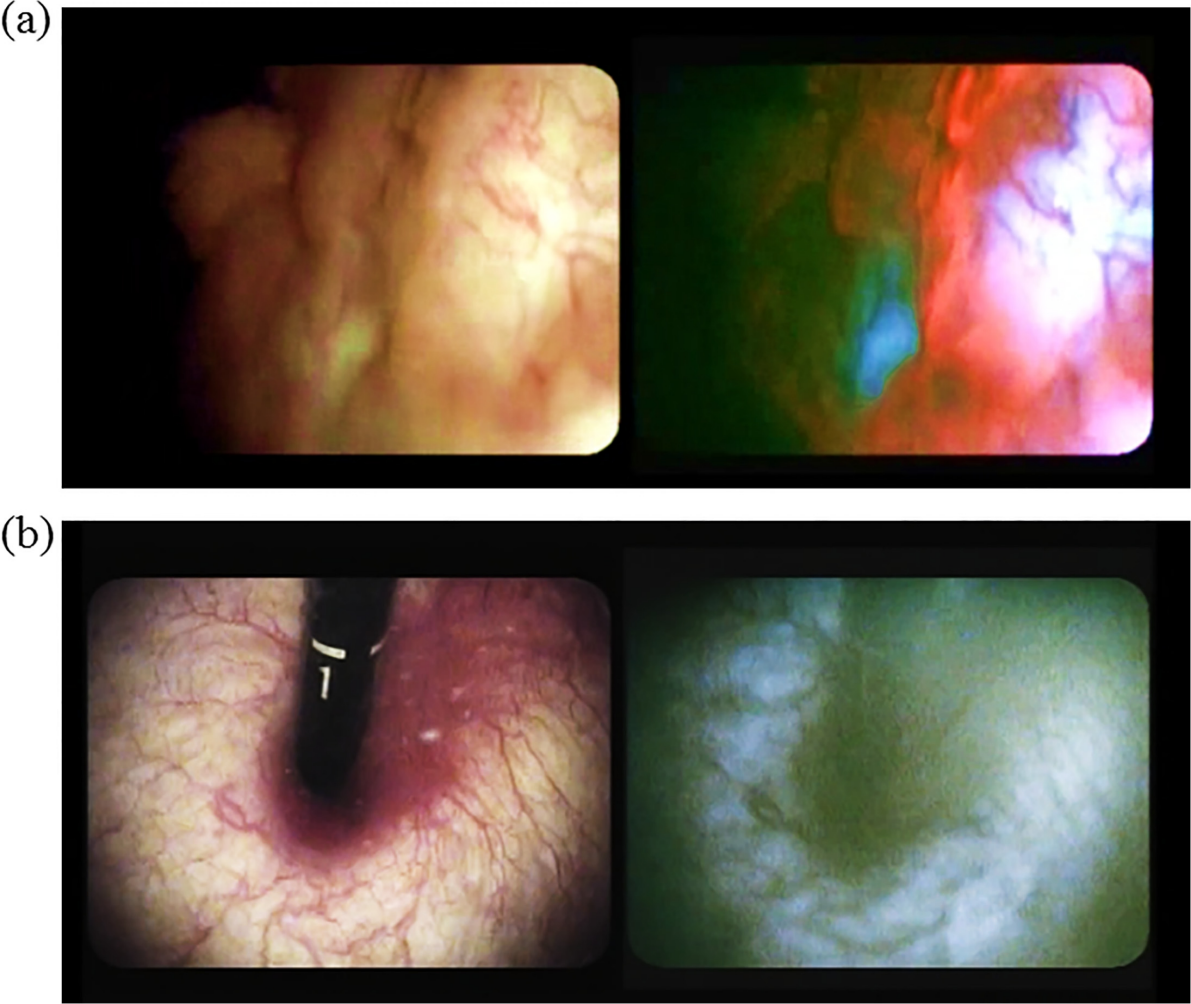
Cystoscopic findings in bladder tumors. (a) White light mode (left side) and blue light mode (right side) images of bladder cancer simultaneously observed using a twin mode monitor. Flat lesions showed red fluorescence. (b) White light mode (left side) and blue light mode (right side) images of the bladder neck using turned back flexible cystoscopy in a vertical direction. No red fluorescence was observed.

Cold-cup biopsy in trigone and bladder neck regions was performed using flexible cystoscopy.

These two regions were inspected with retroflexion flexible cystoscopy in a perpendicular direction. Cold-cup biopsy using rigid cystoscopy was performed in the remaining 5 regions.

We semi-quantitatively divided fluorescent emissions with fluorescent light and abnormalities with white light into 3 categories. According to a clinical study on brain tumors performed by Miyoshi et al. [14], we divided these emissions depending on fluorescence intensity as follows: none (no fluorescent emission), weak (weak fluorescent emission), and strong (strong fluorescent emission).

Furthermore, according to macroscopic findings, we divided the results into three levels as follows: none: no abnormal findings; positive: mild abnormality with no definitive benign or malignant diagnosis; and strongly positive: marked abnormality with a high possibility of malignancy.

These procedures were performed by the same 3 instructors certified by the Japanese Urological Association, and high-level reproducibility was demonstrated in previous reports [15].

### Criteria for diagnostic procedures

Diagnostic accuracy based on a semi-quantitative index was analyzed by comparing the red fluorescence intensity with pathological results according to the General Rules for Clinical and Pathological Studies on Bladder Cancer, Third Edition [16]. We defined macroscopic findings that appeared more than “weak” in fluorescence intensity and “Positive” in white light mode as positives and calculated the appropriate endpoint (predictive accuracy, sensitivity and specificity). The diagnostic capacity was obtained from the area under the receiver operative characteristic curve (AUC). These significant differences were analyzed using Fisher’s exact test (2x2), chi-square test and Wilcoxon rank sum test. All *P*-values<0.05 were considered statistically significant.

## Results

### Pathological results (Table 2)

207 specimens from 30 cases were obtained by transurethral biopsy. We obtained 110 specimens using the conventional PDD system and 97 specimens using the new PDD system.

**Table 2:** Pathological features and fluorescence intensity.

Conventional PDD system in all regions	Fluorescence intensity			Total (# samples)
	none	weak	strong	
Normal epithelium	49	23	2	74
Dysplasia	1	3	0	4
UC G2	1	3	6	10
UC G3	0	4	3	7
UC G3-pTis	4	5	6	15
Total (# samples)	55	38	17	110 (p<0.05^#1^)
New PDD system in all regions	Fluorescence intensity			Total (# samples)
	none	weak	strong	
Normal epithelium	71	15	0	86
Dysplasia	0	3	0	3
UC G2	0	4	1	5
UC G3	0	2	0	2
UC G3-pTis	0	0	1	1
Total (# samples)	71	24	2	97 (p<0.05^#1^)
Conventional PDD system in distal regions	Fluorescence intensity			Total (# samples)
	none	weak	strong	
Normal epithelium	12	11	0	23
Dysplasia	0	1	0	1
UC G2	0	2	0	2
UC G3	0	0	0	0
UC G3-pTis	1	1	2	4
Total (# samples)	13	15	2	30
New PDD system in distal regions	Fluorescence intensity			Total (# samples)
	none	weak	strong	
Normal epithelium	16	9	0	25
Dysplasia	0	2	0	2
UC G2	0	1	0	1
UC G3	0	2	0	2
UC G3-pTis	0	0	0	0
Total (# samples)	16	14	0	30

(#1 Chi-square test)

In addition, we obtained 30 distal bladder specimens each using the conventional PDD system and the new PDD system. Pathological evaluation revealed 47 malignant lesions (22.7%) including 16 lesions of the CIS (7.7%), 7 dysplasia lesions (3.4%) and 160 normal lesions (77.3%). In the distal bladder, we obtained 12 malignant lesions (20.0%), including 4 lesions of the CIS (6.7%), 3 dysplasia lesions (5.0%) and 48 normal lesions (80.0%). A semi-quantitative fluorescence analysis revealed that fluorescence intensity was significantly associated with malignant grade in both PDD systems (*P*<0.05). Fluorescence intensity and the pathological characteristics of the biopsy specimens are shown in Table 2.

### Comparison of diagnostic accuracy (Table 3/Fig 3)

The diagnostic accuracy parameters of each ALA-PDD system, including rates of positive findings, predictive accuracy, sensitivity and specificity, were examined in all biopsy specimens, including those obtained from the distal bladder biopsy. The overall sensitivity, specificity and false-positive rate of the new PDD system were 100%, 82.6%, and 17.4%, respectively. On the other hand, those of the conventional PDD system were 83.3%, 66.2% and 33.8%, respectively. The overall false-positive rate of new PDD system improved to 16.4% when compared with the conventional PDD system. The sensitivity, specificity, and false positive rate of the new PDD system in the distal bladder were 100%, 64% and 36%, respectively. Those of the conventional PDD system in the distal bladder were 85.7%, 52.2% and 47.8%, respectively. The false-positive rate of the new PDD system in the distal bladder improved to 11.8%. Moreover, the overall AUC of the new PDD system was significantly greater compared with that of the conventional PDD system (*P*<0.05). We obtained similar significant results in the distal bladder (*P*<0.05).

**Fig 3:**
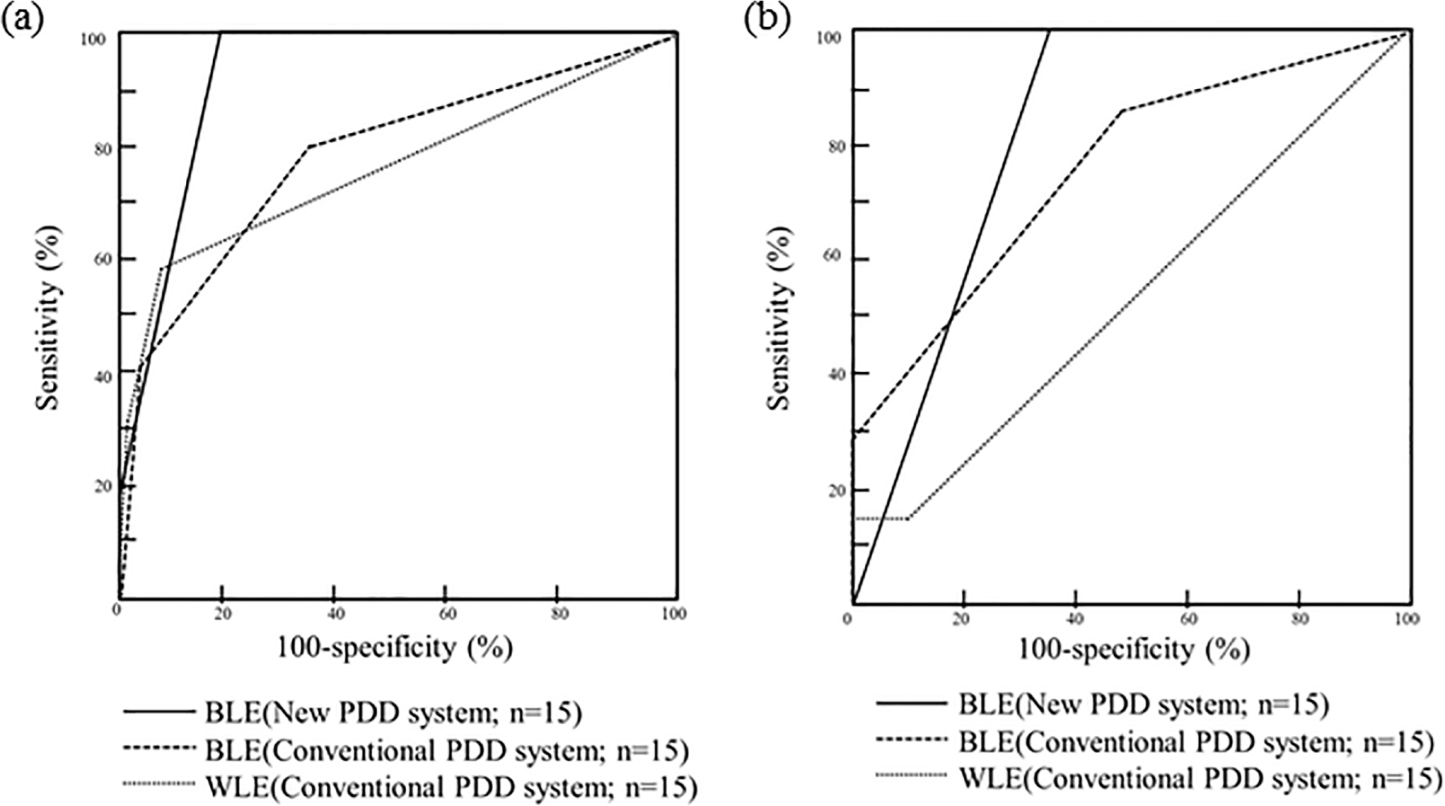
Diagnostic capacity. (a) The AUC of the new PDD system was significantly superior to that of the conventional PDD system in blue light (fluorescence) mode for all regions (*p<0*.*05*). (b) The AUC of new PDD system was significantly superior to that of the conventional PDD system in blue light (fluorescence) mode in the distal bladder (*p<0*.*05*).

**Table 3:** Diagnostic accuracy.

Diagnostic accuracy	Conventional PDD system White light mode (n = 15)	Conventional PDD system Blue light mode(n = 15)	New PDD system White light mode (n = 15)	New PDD system Blue light mode (n = 15)	Conventional PDD system in distal White light mode (n = 15)	Conventional PDD system in distal Blue light mode (n = 15)	New PDD system in distal White light mode (n = 15)	New PDD system in distal Blue light mode (n = 15)
Positive rate (%)	23.6	50.0	8.2	26.8	10.0	56.7	10.0	46.7
Predictive accuracy (%)	80.8	54.5	87.5	42.3	33.3	35.3	66.7	35.7
Sensitivity (%)	58.3	83.3	58.3	100	14.3	85.7	60.0	100
Specificity (%)	93.2	66.2	98.8	82.6	91.3	52.2	96.0	64.0

These results demonstrated that the improvement of diagnostic accuracy in the distal bladder indicated better overall diagnostic accuracy in the new PDD system.

### Adverse events (Table 4)

Of all 30 cases studied, 7 experienced a few adverse events. All adverse events were mild and transient according to the Common Terminology Criteria for Adverse Events version 3.0 (CTCAE). One patient reported a photosensitivity reaction (3.3%), 3 patients had elevated levels of AST and/or ALT (10.0%), and 3 cases experienced nausea/vomiting (10.0%) [17].

**Table 4:** Adverse Events.

Adverse events (Total 30 cases)	Incidence cases (%)	Grade of AEs
	
Photosensitivity	1 (3.3%)	painless erythema
AST / ALT	3 (10.0%)	>1XULN—2.5XULN
Nausea / Vomiting	3 (10.0%)	loss of appetite without alteration
Others	0(0.0%)	in eating habits / 1 episode in 24 hrs
Total cases (%)	7 (23.3%)	

Common Terminology Criteria for Adverse Events v 3.0 (CTCAE)

AST: aspartate aminotransferase, ALT: alanine aminotransferase, ULN: upper limit of normal

## Discussion

This study demonstrated improved diagnostic accuracy for bladder cancer detection using a newly developed PDD system compared with a conventional PDD system. False-positive findings in the distal bladder impair the overall diagnostic accuracy of ALA-PDD [18–23]. The main mechanism behind false-positive findings is considered to be tangent effects in which red-fluorescence is enhanced with observation in the direction of the tangent. This study validated whether the new PDD system improves overall diagnostic accuracy by resolving the tangent effect. Divisi et al. reported that the new PDD system allowed for more accurate detection of lung cancer when compared with the conventional PDD system. These authors demonstrated that the sensitivity and specificity of the new PDD method were 96% and 60%, respectively. The new PDD system therefore resulted in an approximately 30% improvement in sensitivity [24].

Use of the conventional PDD system requires alternation between white light mode to blue light mode on one screen. Furthermore, the blue light mode may provide insufficient illumination to the lesion area for biopsy. Therefore, it is very difficult to obtain appropriate biopsy specimens unless the physician is positioned very close to the lesion. The SAFE-3000 system is equipped with a twin mode PDD system. This system provides simultaneous observation of both white light mode and blue light modes on two screens. This twin mode PDD enables physicians to obtain red-fluorescent tissue using the white light mode. This tool provides sufficient illumination and quick access to the target area, allowing for complete tumor resection. As a result, the new PDD system can prevent photobleaching in red-fluorescent areas.

Furthermore, the SAFE-3000 system equipped with a flexible cystoscope differs from conventional rigid cystoscopy. This means that we can insert the cystoscope smoothly and relieve anxiety and pain for many patients. We have always used rigid cystoscopy for perpendicular observation of the bladder neck and trigone. As a result, there are many false-positive findings due to the tangent effect in the bladder neck and trigone. Moreover, the tangent effect has a negative influence on diagnostic accuracy. However, the SAFE-3000 system is equipped with flexible cystoscopy, which enables perpendicular observation in the bladder neck and trigone. These features can reduce the number of false-positive findings by tangent effect and lead to improved diagnostic accuracy.

In our previous reports, the sensitivity, specificity and false-positive rate for the conventional PDD system were 93.4%, 58.8% and 41.2%, respectively [13]. Thus, high false-positive rates in the conventional PDD system are an intractable clinical problem. Given the diagnostic accuracy of the new PDD system, the sensitivity, specificity and false-positive rate were 100%, 82.6% and 17.4%, respectively. The sensitivity was comparable and the false-positive rate improved compared with that obtained using the conventional PDD system.

Furthermore, in previous comparisons between flexible and rigid fluorescence cystoscopy, flexible cystoscopy was less effective than rigid cystoscopy for tumor detection [25–27]. However, we demonstrated that the AUC of the new PDD system was significantly greater than that of the conventional PDD system for analysis of the distal bladder. The new PDD system with flexible cystoscopy could improve the tangent effect and false-positive findings in the distal bladder.

## Conclusions

Our preliminary results suggest that flexible fluorescence cystoscopy may be more useful than rigid fluorescence cystoscopy for detection of bladder cancer. The use of flexible fluorescence cystoscopy is feasible for avoiding tangent effects, especially in the distal bladder. However, flexible cystoscopy is feasible for diagnosis, but not for surgery. Further randomized studies with larger populations are required.

## Supporting Information

S1 File Protocol in this study. This study was carried out with approval of the Ethics Committee of Kochi Medical School. All participates were informed about aim and adverse events of this study.
(DOCX)
