# Supplementary material for: The Utility of a Flexible Fluorescence-Cystoscope with a Twin Mode Monitor for the 5-Aminolevulinic Acid-Mediated Photodynamic Diagnosis of Bladder Cancer
Source: PLoS One. 2015 Sep 2;10(9):e0136416. doi: 10.1371/journal.pone.0136416 (PMC4558053; doi:10.1371/journal.pone.0136416)
Supplement: S1 File — This study was carried out with approval of the Ethics Committee of Kochi Medical School. All participates were informed about aim and adverse events of this study. (DOCX) [file pone.0136416.s001.docx]

**Supporting information**

**Background**

Bladder cancer is the second most frequent malignant tumor and has an annual incidence of over 60,000, 130,000, and 17,000 in Europe, the United States, and Japan, respectively. Approximately 70% of cases involve non-muscle invasive bladder cancer treated with transurethral resection of bladder tumor (TURBT). However, non-muscle invasive bladder cancer has a high recurrence rate after TURBT. It is thought that residual lesions such as minute or flat lesions play a large role in the high recurrence rate. Methods such as 5-aminolevulinic acid (ALA)-mediated photodynamic diagnosis (PDD) provided good visualization for these minute and flat lesions and improved diagnostic accuracy. The ALA-PDD system was approved as a diagnostic method for non-muscle invasive bladder cancer in Europe and the United states.

**Photodynamic diagnosis (PDD)**

5-Aminolevulinic acid (ALA) is a naturally occurring metabolite that is a precursor of porphyrin in heme biosynthesis. Exogenous ALA leads to accumulation of the potent photosensitizer PpIX in mitochondria. PpIX is known to accumulate more in malignant and proliferating tissues than in normal tissues. In this way, PpIX selectively accumulates at a significant level in tumor cells. PpIX is an effective photosensitizer and fluorescent substance in heme biosynthesis. 5-Aminolevulinic acid (ALA)-mediated photodynamic diagnosis (PDD) is used widely in various cancers, including bladder cancer.

**The objective of this study**

We reported the feasibility of ALA-PDD for bladder cancer. ALA-PDD significantly improved diagnostic accuracy and intraoperative detection of bladder cancer, in particular for flat lesions such as dysplasia and CIS. However, there were many false-positive findings in the distal bladder (trigone and neck portions of bladder). The specificity in these regions was significantly lower than in the other portions of the bladder. The main reason for false-positive findings in the trigone and neck areas is that red fluorescence is enhanced with observation in the direction of the tangent, even if the tissue is normal. This phenomenon is called the tangent effect. This tangent effect is an important problem for false-positive findings in ALA-PDD. A flexible scope can directly confront the cervix tissue and therefore avoid the tangent effect. In the new PDD system, a flexible scope was used to observe the bladder cervix and a processor was used (SAFE-3000, PENTAX, HOYA Co, Tokyo), which can simultaneously display both a white light image and a fluorescent image on a video monitor (twin mode). With systems where only one image (white or fluorescent light) can be displayed at a time, surgeons have to use their memory to compare the two images when switching back and forth. Utilizing the twin mode, both images can be compared on the same monitor, which is expected to result in more accurate diagnoses. In this study, we retrospectively evaluated the feasibility of ALA-PDD using the SAFE-3000 system (new PDD system) and determined whether the flexible cystoscopy improves false-positive findings and diagnostic accuracy in the trigone and neck regions of the bladder.

**Clinical trial medicine**

ALA hydrochloride (COSMO BIO, Tokyo, Japan) was dissolved in 50 ml of 5% glucose solution. Patients were orally administered 1.0 g of ALA solution two to three hours prior to intraoperative surveillance before anesthesia.

**Clinical trial equipment**

A fluorescence endoscope consisting of a processor (SAFE-3000) and a flexible scope (EB-1970AK, PENTAX) was used. A system diagram of the processor is shown in Fig. 1. It has a xenon lamp as a white light source (300 W) with a semiconductor laser (wavelength of 408 nm) with a power at the top of the scope of 20 to 40 mW. The wavelength of the laser is close to that of the absorption maximum of PpIX at 405 nm. A series of processes (absorption by PpIX, excitation of PpIX, and fluoresce from PpIX) can then be detected with high efficiency. The spectrum of the fluorescence has a broad peak of approximately 635 nm; thus, fluorescence images can be observed with a charge coupled device image processor (CCD). A filter is mounted on the CCD in order to cut the strong blue light reflected by organ surfaces. RGB signals from the CCD can be independently tuned. The sensitivity to red color was maximized and that to green was minimized in the present report, which generated red fluorescent images of lesions on a bluish background. A white light image and a fluorescent image were simultaneously displayed side-by-side on a monitor (twin mode). The side-by-side image is composed of a white light image captured in the first 1/60 second and a fluorescent image captured in the next 1/60 second. In the first 1/60 second, white light is emitted through a shutter that opens for 1/60 second. In the next 1/60 second, a blue laser light is emitted by switching on the laser chip for 1/60 second. These white and blue light beams of different orientations are led to a beam splitter to be focused on the edge of an optical fiber. The light is transmitted in the optical fiber and emitted from the top of the scope. Then, the organ surface to be observed is alternatively illuminated by white and blue light every 1/60 second. Images of the surface are taken by the CCD. A white light image from the first 1/60 second and a fluorescent image from the following 1/60 second are held in a memory chip ,and each image is simultaneously displayed for 1/30 second on the monitor to produce video images at 30 frames/second. When the white light video or fluorescent video is selected to be displayed, the beam splitter is ejected from the beam line and the objective light source is turned on to produce video images at 30 frames/second. The diameter of the scope is 6.3 mm at the top and 2.8 mm at the channel. The bending angle at the top is 130 to 180 degrees. A tube for injection and ejection of water was connected to manipulate part of the scope and operated with a button. Forceps for biopsy (FB-15C-1; Olympus, Tokyo) and a hemostasis electrode (CD-6C-1; Olympus) were used. It should be noted that the EB-1970AK scope is designed for the bronchus and was used for bladder cancer observation in this study with the permission of the ethics board of Kochi University.

**Intraoperative procedure**

Transurethral observation of the bladder was performed under white light and fluorescence light guidance. The specimens with fluorescent emission or with suspicious abnormalities under white light guidance were systematically harvested from 7 bladder regions (left, posterior, right walls, trigone, bladder neck and prostatic regions of the urethra). Cold-cup biopsy in the trigone and bladder neck regions was performed using flexible cystoscopy. These two regions were inspected with turn-back flexible cystoscopy in a vertical direction. Cold-cup biopsy was performed in the remaining 5 regions using rigid cystoscopy. We semi-quantitatively divided the fluorescent emissions with fluorescent light and abnormalities with white light into 3 categories as follows. According to a clinical study on brain tumor performed by Miyoshi et al., we divided the results depending on fluorescence intensity in the following manner: none (no fluorescent emission), weak (weak fluorescent emission), and strong (strong fluorescent emission). Furthermore, we divided the results into three levels as follows according to macroscopic findings: none: no abnormal finding; positive: mild abnormality with no definitive benign or malignant diagnosis; and strongly positive: marked abnormality with a high possibility of malignancy. These procedures were performed by the 3 same instructors certified by the Japanese Urological Association, and high-level reproducibility was demonstrated in previous reports.

**Primary endpoint**

**Diagnostic accuracy**

The evaluation of diagnostic accuracy in this study is the comparison between fluorescence intensity and pathological results according to general rules for clinical and pathological studies of bladder cancer. We used a statistical analysis of diagnostic accuracy (sensitivity and specificity) compared to the conventional PDD system as an historical control.

**Secondary endpoint**

**Frequency of adverse events**

We assessed the occurrence of early and late adverse events. The evaluation of adverse events was in accordance with the Common Terminology Criteria for Adverse Events Ver 3.0 (CTCAE-V3.0).

**Statistical analysis**

Diagnostic capacity was obtained from the area under the receiver operative characteristic curve (AUC). These significant differences were analyzed using Fisher’s exact test (2x2), chi-square test and Wilcoxon rank sum test. All *P*-values<0.05 were considered statistically significant.

**Collaborating research institutes**

SBI Pharmaceuticals Co., Ltd (Supplied 5-aminolevulinic acid)

R&D Center, HOYA Corporation (Supply and maintenance of SAFE-3000 and EB-1570AK)

**Scheduled trial subjects**

The total number of scheduled cases is 15. A total of 15 cases were retrospectively compared with the conventional PDD system as a historical control and with conventional white light examination. There were no statistically significant differences among patients with regards to tumor characteristics.

**Inclusion criteria**

Patients with non-muscle invasive bladder tumor considered as an indication for TURBT were enrolled in this clinical trial after having provided written informed consent.

**Exclusion criteria**

The patients who had liver or renal function abnormality or porphyria were excluded from this clinical trial because they had the potential for severe adverse events. The patients whom doctors considered inappropriate for study were also excluded from this clinical trial.

**Study duration**

This clinical trial is conducted from December 2011 to December 2012.

**Discontinuance criteria**

In the event of any of the following conditions, this clinical trial is stopped and reconsidered the necessity of continuing.

1. In case of a severe adverse event (grade 3/4 by CTCAE-V3.0)
2. In case of an adverse event that makes it difficult to continue this clinical trial.
3. In case of a post-onset revelation that excludes a patient from this clinical trial.
4. In cases where the subject is required to stop.
5. In cases where doctors consider it to be difficult for an enrolled patient to continue.

**Informed consent**

This study was performed after written informed consent was obtained from patients.

The statement of informed consent was approved by the ethical committee.

Involvement of companies

This clinical trial is conducted as a cooperative study (SBI Pharmaceuticals Co., Ltd/ R&D Center, HOYA Corporation).
